# Recovery from Repeated Sudden Hearing Loss in a Patient with Takayasu's Arteritis Treated with Hyperbaric Oxygen Therapy: The First Report in the Literature

**DOI:** 10.1155/2017/3281984

**Published:** 2017-09-10

**Authors:** Massimo Ralli, Antonio Greco, Vincenzo Falasca, Giancarlo Altissimi, Mario Tombolini, Rosaria Turchetta, Sara de Fazio, Marco de Vincentiis, Giancarlo Cianfrone

**Affiliations:** ^1^Department of Oral and Maxillofacial Sciences, Sapienza University of Rome, Rome, Italy; ^2^Department of Sense Organs, Sapienza University of Rome, Rome, Italy

## Abstract

Hearing loss has been rarely reported in Takayasu's arteritis, may present as Sudden Sensorineural Hearing Loss, and usually responds well to corticosteroid therapy. Hyperbaric Oxygen Therapy is commonly used as a supplementary treatment to corticosteroids for Sudden Sensorineural Hearing Loss. We present the case of a 36-year-old woman with Takayasu's arteritis who had two episodes of sudden hearing loss involving one ear at a time with an 11-month delay between each episode. During the first episode, the patient was treated with high-dose intramuscular corticosteroids with a temporary improvement of auditory threshold that deteriorated 14 days after cessation of therapy. In the second episode, Hyperbaric Oxygen Therapy was associated with corticosteroids, with improvements in both ears, including the one that was unresponsive in the long term to previous pharmacologic therapy. In this case, Hyperbaric Oxygen Therapy could have acted synergically with corticosteroids playing a role in hearing restoration.

## 1. Introduction

Takayasu's arteritis (TA), also known as aortitis syndrome, is a vasculitis more prevalent in women of reproductive age that mainly affects large elastic arteries with symptoms caused by organ ischemia, aneurysm formation, and inflammation [1]. Clinical features usually reflect limb or organ ischemia that follow gradual stenosis of the involved arteries [2, 3].

Hearing loss (HL) has been rarely reported in patients with TA. HL can be stable or fluctuating, involves mainly high frequencies, may present as a Sudden Sensorineural Hearing Loss (SSHL), and usually responds well to corticosteroid therapy. HL in TA is often diagnosed as idiopathic SSHL.

Hyperbaric Oxygen Therapy (HBOT) involves breathing pure oxygen and is commonly used as a supplementary treatment to corticosteroid treatment for SSHL by increasing the supply of oxygen to the inner ear and brain. Current research supports the role of HBOT in SSHL if used within two weeks from the onset of HL, while there is no evidence that HBOT can help after that time [4, 5]. A recent paper on 167 SSHL cases reported that HBOT, if performed appropriately, could be able to improve hearing in many cases unresponsive to initial corticosteroid therapy [6].

There are no studies in the literature that report the use of HBOT for the treatment of HL in patients with TA. The aim of this study is to present a case of a TA patient with two episodes of SSHL which occurred at different times treated with HBOT in addition to corticosteroid therapy.

## 2. Case Report

A 36-year-old woman affected by TA was treated in the Otolaryngology Department of the Policlinico Umberto I Hospital, Sapienza University of Rome, following two episodes of sudden hearing loss with continuous low-pitch tinnitus without vertigo and associated neurovegetative symptoms. The first episode occurred in April 2016 and involved the right ear; the second occurred in March 2017 and involved the left ear.

**Figure 1:**
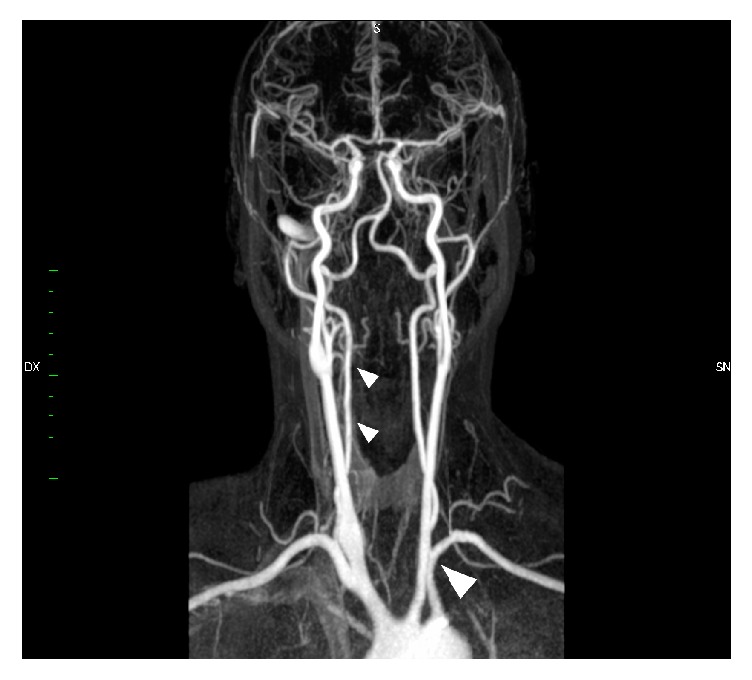
Magnetic Resonance Angiography (MRA) showing narrowing of the common carotid arteries bilaterally and the left subclavian artery (large arrowhead). Vertebral and intracranial carotid arteries were normal bilaterally.

Diagnosis of TA was posed in 2001 following the criteria of the American College of Rheumatology [7] and was based on the onset of asthenia, sideropenic anemia, asymmetry of radial arterial pulsation, and amaurosis fugax. Laboratory tests revealed increased inflammatory markers (erythrocyte sedimentation rate, C-reactive protein) and epiaortic ultrasound showed a significant stenosis of the left subclavian artery and right common carotid artery and a nonsignificant stenosis of abdominal aorta, renal artery, and mesenteric artery. Magnetic Resonance Angiography (MRA) confirmed diagnosis (Figure 1). High-dose steroid therapy (prednisone 50 mg/day) and methotrexate (15 mg/day) were administered and a progressive stabilization of clinical and laboratory findings was obtained in the following 15 years. Maintenance steroid therapy was initiated (0.3 mg/kg methylprednisolone/day) and continued until present time.

After admission following the first episode of SSHL in April 2016, patient underwent a complete ENT examination with otoscopy, Pure Tone Audiometry (PTA), Immittance test, Auditory Brainstem Response (ABR), Distortion-Product Otoacoustic Emissions (DPOAE), MRA, and Ultrasound.

**Figure 2:**
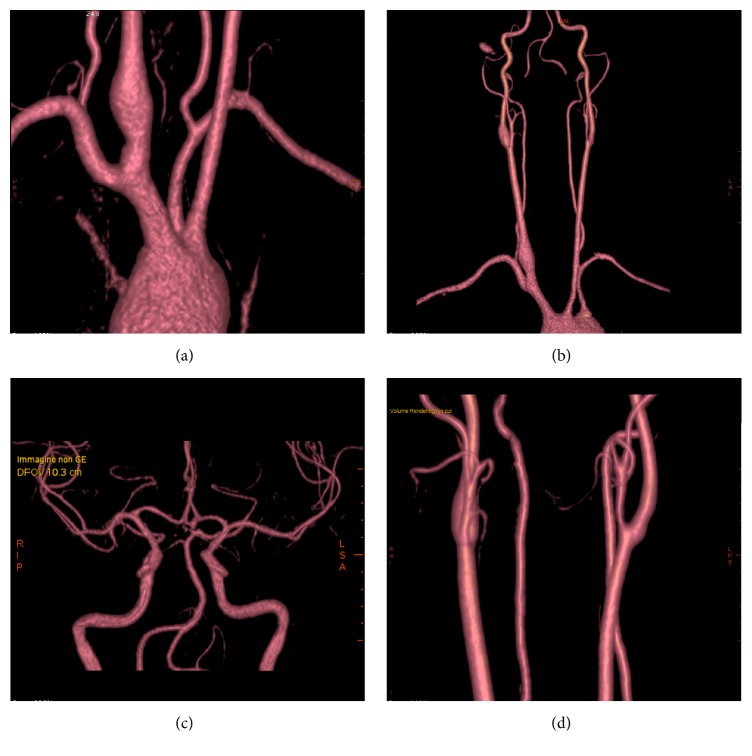
Volume-rendered reconstructions from Magnetic Resonance Angiography examination showing diffuse narrowing of the left subclavian artery (a) and common carotid arteries bilaterally (b). Normal caliber was found for intracranial arteries ((c)-(d)).

**Figure 3:**
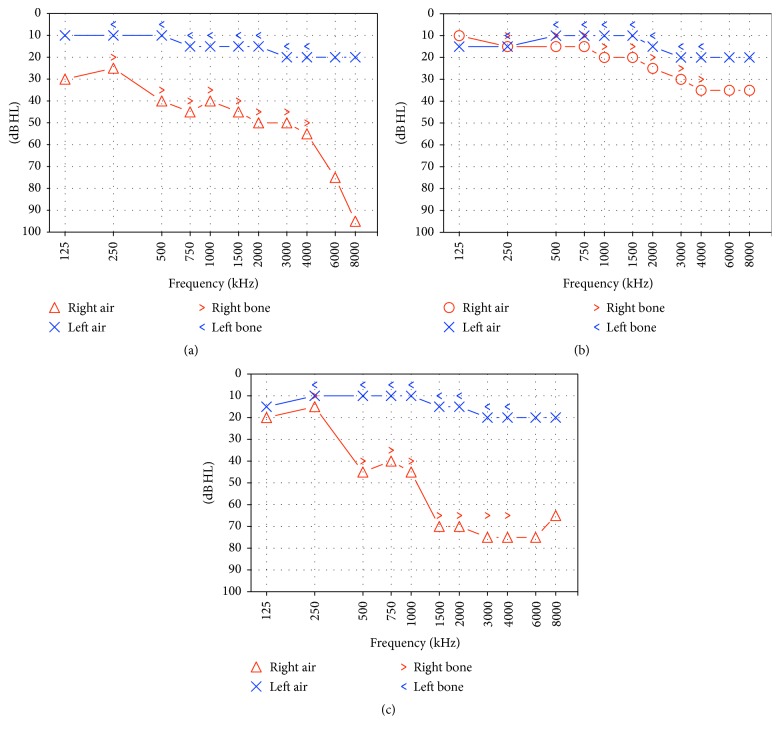
PTA in our patient recorded one, three, and 14 days after onset of Sudden Sensorineural Hearing Loss in the right ear (first episode). (a) Pure Tone Audiometry recorded one day after hearing loss onset showing a moderate sensorineural hearing loss in the right ear with an average auditory threshold of 49.54 dB HL and normal hearing threshold in the left side. (b) Pure Tone Audiometry recorded three days after hearing loss onset; hearing in the right side significantly improved (average threshold: 23.2 dB HL). (c) When recording Pure Tone Audiometry 14 days after sudden hearing loss onset, and 4 days after cessation of high-dose corticosteroid therapy, hearing in the right ear returned to previous levels with an average threshold of 54.09 dB HL.

MRA showed a significant stenosis of the left subclavian artery and common carotid arteries bilaterally and a nonsignificant stenosis of abdominal aorta, renal artery, and mesenteric artery. Volume-rendered reconstructions from MRA are shown in Figure 2. Ultrasound showed no vascular changes in the epiaortic vessels compared to the previous examinations. Positron Emission Tomography showed worsening of the vascular inflammatory state, mainly involving the common, external, and internal carotid arteries and vertebral arteries. PTA showed moderate sensorineural hearing loss in the right ear with an average PTA threshold of 49.54 dB HL and normal hearing in the left side (Figure 3(a)). Weber test was lateralized to the left at all frequencies. Immittance test was within normal range. ABR revealed in the right ear the absence of the 1st wave and an increased hearing threshold (80 dB).

The patient was treated with high-dose intramuscular steroid therapy (betamethasone 8 mg/day for 10 days), in addition to ongoing maintenance steroid therapy. Her audibility significantly improved three days after the beginning of treatment (average threshold in the right ear: 23.2 dB HL) (Figure 3(b)) but deteriorated close to previous level (average threshold in the right ear: 54.09 dB HL) 14 days after cessation of therapy, as shown in Figure 3(c). Hearing remained stable over the following months.

**Figure 4:**
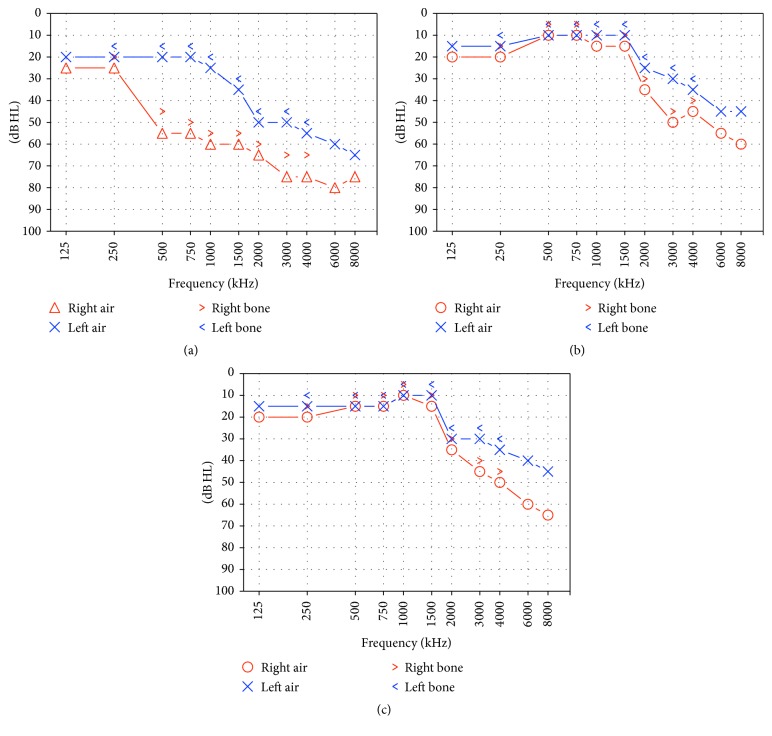
Pure Tone Audiometry recorded one, 14, and 60 days after onset of sudden hearing loss in the left ear (second episode). (a) Pure Tone Audiometry confirmed hearing loss in the left ear, with an average threshold of 38.18 dB HL; threshold in the right ear was unmodified compared to the exam performed 14 days after cessation of previous treatment (56.09 dB HL); (b) Pure Tone Audiometry recorded 14 days after hearing loss onset showed a significant improvement of hearing loss in the left ear (average: 22.72 dB HL) but also in the right ear (average: 30.45 dB HL); (c) Audiometry 60 days after left sudden hearing loss onset showed no significant differences compared to previous exam, with preserved hearing threshold in both ears.

Several months later, in March 2017, the patient was admitted to our Department complaining of a new episode of SSHL which occurred in the left ear. PTA confirmed a new onset SSHL in the left ear, with an average PTA threshold of 38.18 dB HL; threshold in the right ear was unmodified compared to the exam performed 14 days after cessation of previous treatment (56.09 dB HL) (Figure 4(a)). The patient was already undergoing maintenance steroid therapy (0.3 mg/kg methylprednisolone/day). The patient was treated with high-dose intramuscular steroid therapy (betamethasone 8 mg/day for 10 days) accompanied by 16 sessions of HBOT (1/day, 6 days/week) started two days after the SSHL episode. PTA recorded 2 weeks after SSHL onset showed a significant improvement of HL in the left ear (average: 22.72 dB HL) but also in the right ear (average: 30.45 dB HL) (Figure 4(b)). Hearing thresholds in both ears remained stable over the following two months (Figure 4(c)).

## 3. Discussion

### 3.1. Takayasu's Arteritis and Hearing Loss

In this patient, hearing loss presented as SSHL in two episodes involving one ear at a time, with an 11-month delay between each other and with different paths of hearing recovery. The first episode, occurring in the right ear, was treated with corticosteroid therapy only and showed hearing improvement during therapy that deteriorated soon after cessation of the therapy. The second (left ear) was treated with corticosteroid therapy and HBOT; this therapy induced a significant improvement in the affected ear and, interestingly, also in the contralateral side. In addition, hearing thresholds in both ears did not change for a period of two months after completion of the therapy.

There are only a few cases in the literature that reported an association of TA with HL [8–10]. HL in TA may present as SSHL and is usually diagnosed and treated as an idiopathic SSHL. However, HL associated with TA arteritis is often progressive and fluctuates during treatment; it may have a good response to corticosteroid therapy but may also persist despite corticosteroid therapy [11]. A progressive fluctuating bilateral asymmetric SNHL that develops over several weeks to months is a common finding in autoimmune disorders [12, 13].

The etiology of hearing deficits in TA is unknown [14, 15]. A connection with the autoimmune pathophysiology of TA has been hypothesized due to the elevation of serum immune complexes in TA that deposit in the inner ear [14], hypercoagulability in response to the arterial disease [16], and the good response to anti-inflammatory therapy with corticosteroids. Although TA involves medium and large caliber arteries, Maruyoshi speculated that HL in TA could have a vascular background based on reversible circulatory disturbances due to vasculitis and/or some autoimmune pathogenesis in the inner ear, especially in hair cells [11].

### 3.2. Effects of Steroid Therapy on Hearing Loss in Takayasu's Arteritis

Many authors confirmed the beneficial effects of steroid administration on HL in TA; therefore steroids should be used as first-line therapy in this condition [9, 14, 15]. However, steroid therapy must be administered chronically to control TA symptoms; the interruption of steroid treatment has been reported to exacerbate the hearing deficit [15]. Intratympanic injection of steroids may be an alternative treatment to systemic therapy.

One of the first authors to report HL in TA was Kanzaki in 1981, who described a beneficial effect of prednisolone [14]. Murofushi et al. reported a case of a 44-year-old woman with fluctuating sensorineural HL in the high frequencies suffering from TA who was treated with intratympanic injection of dexamethasone at the time of worsening of hearing [17].

Kunihiro et al. reported 5 cases of TA patients with HL in which the degree of hearing loss correlated well with the erythrocyte sedimentation rate. All patients responded well to corticosteroid therapy, supporting the hypothesis that inner ear manifestations of the disease arose from similar mechanisms as those of systemic inflammatory process [9]. In 2005 Maruyoshi et al. reported a case of a 49-year-old woman with TA who experienced sudden hearing loss five years before diagnosis of TA, treated with systemic corticosteroid therapy. After cessation of the therapy, HL worsened. After TA diagnosis, the patient was treated with daily prednisolone (0.3 mg/kg/day) with significant improvement in hearing threshold in the right ear, while no changes were found in the contralateral ear [11].

TA has been reported to be associated with Cogan's syndrome [18], a rare disorder of unknown origin characterized by inflammatory eye disease (74% of the cases) with interstitial keratitis and vestibuloauditory symptoms [19]. In this case, ocular and vestibular symptoms were never reported despite the long history of TA in the patient; furthermore, auditory involvement is always severe and frequently progresses within several hours or days to total deafness [20]. Therefore, Cogan's syndrome was ruled out in this case.

### 3.3. Possible Role of Hyperbaric Oxygen Therapy in Treating Hearing Loss in Takayasu's Arteritis

In this patient, we found a significant improvement of hearing threshold in both ears with HBOT performed in conjunction with high-dose corticosteroid therapy after the second episode of SSHL. Interestingly, HBOT induced a rapid increase in hearing threshold in both ears, more marked in the right ear in which hearing significantly decreased 11 months prior to therapy, and had shown to be unresponsive in the long term to steroid therapy performed immediately after HL onset. A possible explanation of this finding could be attributed to the vascular effects of hyperbaric therapy, such as enhancing oxygen, glucose, and adenosine triphosphate delivery to ischemic tissues, and vasodilation. However, the selectivity of TA for medium and large arteries raises questions about effectiveness of such therapy in small vessels such as in the inner ear; our findings could support the involvement of inner ear microcirculation in HL presenting in TA patients.

Although the exclusive role of HBOT in this patient cannot be documented since it has been performed in addition to steroid therapy and no long term follow-up is available, the significant improvement on HL in both sides, including the one in which HL presented over 11 months prior to HBOT, is certainly worth discussion. In this case, HBOT could have worked synergically with steroid therapy and amplified treatment results in the long term.

Current findings certainly require further studies on larger samples; however, they may suggest, when no individual contraindication is present, the use of HBOT even several months after HL onset in patients with a diagnosis of TA.
